# Threshold somatic cell count for delineation of subclinical mastitis cases

**DOI:** 10.14202/vetworld.2018.789-793

**Published:** 2018-06-12

**Authors:** P. V. Jadhav, D. N. Das, K. P. Suresh, B. R. Shome

**Affiliations:** 1 Department of Animal Genetics and Breeding, College of Veterinary and Animal Sciences, Udgir - 413 517, Latur, Maharashtra, India; 2 Principal Scientist, South Regional Station of National Dairy Research Institute, Bengaluru - 560 030, Karnataka, India; 3 Principal Scientist, ICAR-National Institute of Veterinary Epidemiology and Disease Informatics, Yelahanka - 560 064, Bengaluru, Karnataka, India

**Keywords:** discriminate function, mastitis, receiver’s operating characteristic curve, somatic cell count, threshold

## Abstract

**Aim:**

Somatic cell count (SCC) is the most widely used single reliable indicator of udder health. The present study was carried out with an objective to find the exact threshold of SCC.

**Materials and Methods:**

Milk samples collected from a total of 214 Holstein Friesian crossbred dairy animals were subjected to bacterial DNA extraction and SCC estimation by digital PortaCheck. California Mastitis Test and polymerase chain reaction based on amplification of organism using reported primers were performed to diagnose subclinical mastitis. Receiver’s operating characteristic (ROC) curve analysis and discriminate function analyses were performed using SPSS 18 software.

**Results:**

ROC curve analysis represented that the area under the curve was 0.930 with the standard error of 0.02. Results indicated that 93% of the case could be correctly predicted as mastitis infected using SCC as a marker (p<0.001). At cut score level of 282 000 cells/ml, 285,000 cells/ml and 288,000 cells/ml, sensitivity remained 92.6% and specificity augmented as 86.3%, 87.2%, and 88%, respectively. At SCC value of 310,000 cells/ml of milk, sensitivity and specificity were optimal, namely, 92.6% and 91.5%, respectively. The function fitted demonstrated 89.2% accuracy with p<0.001. The functions at group centroids were −0.982 and 1.209, respectively, for normal and mastitis-infected animals and log_SCC value was the most important factor contributing 38.30% of the total distance measured.

**Conclusion:**

Our study supports that the threshold value to delineate subclinical mastitis case from the normal is 310,000 somatic cells/ml of milk and a model so fitted using the variable SCC can be successfully used in field for the diagnosis of subclinical cases of mastitis which otherwise would be difficult to differentiate based on clinical signs.

## Introduction

Amid the economic diseases of dairy industries, mastitis paves its way notably. A better strategy to battle with it would be the prevention of occurrence. Fortunately, advance researches have gifted us with an alarm that presages the onset of mastitis. The best indicator of the onset of inflammation in the mammary gland is the shedding of somatic cells in milk. It is the most widely used single reliable indicator of udder health and is a useful predictor of intramammary infection (IMI) [1]. Fanatical monitoring of milk somatic cell count (SCC) can help the livestock owners to set a check post for entry of disease in the herd. Somatic cells are mainly milk-secreting epithelial cells and white blood cells including neutrophils, monocytes, macrophages, and lymphocytes.

The high number of somatic cells is found because the mammary epithelial cells mount defense mechanism against invading pathogens by detecting their ligands and initiate appropriate immune responses [2]. Although the somatic cells in milk increase in IMI, they may in few numbers be normally secreted in the milk regularly. The skill lies in demarking this exact threshold that pushes the animal in the subclinically infected category. This study will help define a threshold for SSC to alarm a subclinical case of IMI.

The present study was carried out with an objective to determine the cutoff value of SCC to delineate subclinical mastitis cases aligned with the normal ones.

## Materials and Methods

### Ethical approval

The approval from Institutional Animal Ethics committee was not required as no invasive method that would give pain to animals was performed.

### Experimental material

The study was conducted in a total of 214 Holstein Friesian (HF) crossbred dairy animals maintained by farmers located in both Bengaluru urban and rural districts. About 35-40 ml of composite milk sample was collected from each lactating animal to estimate the SCC and to extract the bacterial DNA.

### Data recorded

Data were recorded on the season of sample collection, parity of the animal, stage of lactation, milk production capacity, and genetic composition (crossbred or graded). Observations were made regarding the farm effect, udder hygiene status, stall hygiene status, and method of milking followed (hand milking/machine milking).

### Screening of milk samples

SCC was estimated on the same day within 12 h of milk collection using digital SCC (PortaCheck). California Mastitis Test (CMT) reflects the SCC level quite accurately [3], and hence, it was used to screen the animals. Observations were recorded as normal, slight thickening or gel formation after adding the chemical. Samples showing slight thickening or gel formation were regarded as subclinical case. Sever gel formation cases were excluded from the experiment.

All the milk samples were subjected to bacterial DNA extraction as indicated by Tarate *et al*. [4]. Reported primers by Shome *et al*. [5] for the identification of five bacterial species, namely, *Staphylococcus aureus*, *Staphylococcus epidermis*, *Streptococcus* *agalactiae*, *Streptococcus dysgalactiae*, and *Escherichia coli* were used to amplify the region of interest, and the samples were categorized as normal or infected depending on the presence or absence of the bacteria. Further for receiver’s operating characteristic (ROC) curve analysis, the samples showing slight thickening and gel formation in CMT also showing the presence of one or the other above-mentioned bacteria were determined as subclinical. ROC curves were used to interpret sensitivity and specificity levels and to determine related cut scores of milk SCC in affected animals.

### Statistical analysis

An attempt was also made to develop a functional model which could help to discriminate healthy and infected animals. ROC curve analysis and discriminate function analysis were performed using SPSS 18 software.

The linear discriminate function model considered was as follows:

D=a+b_1_M_1_+b_2_M_2_+b_3_M_3_+……+b_10_M_10_

Where, i = 1, 2, 3,…., 10

D - Total discriminant score for normal and infected animals

M_1_ - Standardized log somatic cell count (log_SCC = log_2_(SCC) + 4)

M_2_ - Standardized stage of lactation of the animals under study (I, II, or III)

M_3_ - Standardized indicator if the samples collected in the rainy season (1 if yes and 0 if no)

M_4_ - Standardized indicator if the samples collected in the winter season (1 if yes and 0 if no)

M_5_ - Standardized parity of the animals (1, 2, 3, 4, 5, and above)

M_6_ - Standardized stall hygiene score (1 if daily cleaning of the animal house done with disinfectant, 2 if daily cleaning of the animal house done with water only, 3 if occasional cleaning done, and 4 if no cleaning done at all)

M_7_ - Standardized udder hygiene score (1 if daily cleaning of the animal house done with disinfectant, 2 if daily cleaning of the animal house done with water only, 3 if occasional cleaning done, and 4 if no cleaning done at all)

M_8_ - Standardized method of milking indicator (1 if hand milking and 0 if machine milking)

M_9_ - Standardized indicator of genetic group (1 if graded HF and 0 if crossbred HF)

M_10_ - Standardized test day milk yield in kg

a - Is a constant and

b_i_ - Is the unstandardized canonical discriminant function coefficients.

## Results

**Figure-1:**
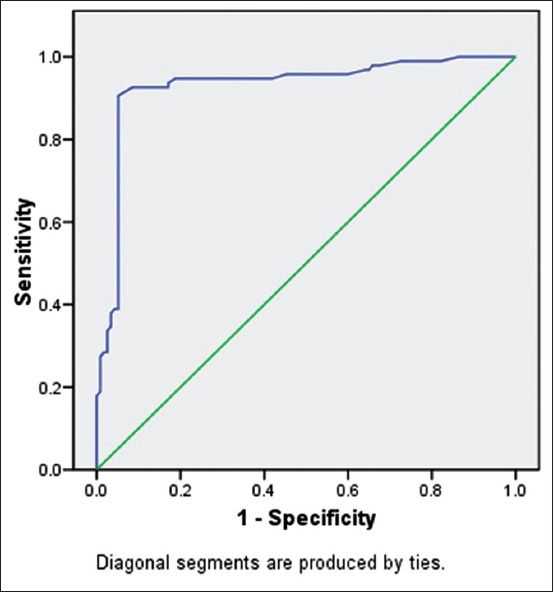
Receiver’s operating characteristic curve for representing the diagnostic performance of somatic cell count (in millions) as an indicator of mastitis.

ROC curves are the generalization of the set of potential combinations of sensitivity and specificity possible for predictors [6]. Analysis represented that the area under the curve was 0.930 with the standard error of 0.02. The results indicated that 93% of the case could be correctly predicted as mastitis infected using SCC as a marker (p<0.001). The graphical representation of the ROC curve using SCC as a predictor of the mastitis condition demonstrated in Figure-1. The values of sensitivity and specificity at cut score level of 276,000 cells/ml of milk were 92.6% and 82.9%, respectively. At cut score level of 282,000 cells/ml, 285,000cells/ml, and 288,000cells/ml, sensitivity remained 92.6%; however, specificity augmented with values as 86.3%, 87.2%, and 88%, respectively (Table-1). At cut score level of 310,000 cells/ml of milk, the values of sensitivity and specificity were optimal, namely, 92.6% and 91.5%, respectively. This went on reducing with the increase in the SCC. The results indicated that 310,000 somatic cells/ml of milk should be the threshold value to differentiate the subclinically affected animals from the normal ones.

**Table-1:** Diagnostic performance of SCC as an indicator of subclinical mastitis.

SCC cutoff (millions)	Sensitivity	Specificity
0.2775	0.926	0.829
0.2820	0.926	0.863
0.2845	0.926	0.872
0.2875	0.926	0.88
0.3100	0.926	0.915

SCC=Somatic cell count

For discriminate function analysis, among all the factors considered, it was found that log_SCC, stage of lactation, rainy season, stall hygiene score, udder hygiene score, and method of milking contributed significantly (p<0.01) to calculate the difference between the normal and the infected animals. Eigenvalue for the above factors was calculated as 1.273, and the overall Wilks’ lambda value was 0.440. Chi-square value was 167.549 at 10 degrees of freedom and the model significantly (p<0.01) classified 90.0% of original grouped cases correctly. The results revealed that log_SCC value was the most critical factor in discriminating normal and mastitis-infected animals and contributed 38.30% of the total distance measured. Percentage contribution of hand milking, udder hygiene score, stall hygiene score, rainy season, and stage of lactation were 28.87, 24.55, 4.54, 2.52, and 1.22, respectively (Table-2).

**Table-2:** Contribution of individual variable to the total distance measured.

Variables	Standardized canonical discriminant function coefficients	Contribution (%)
Log_SCC	1.003	38.30
Stage of lactation	0.032	1.22
Rainy	−0.066	2.52
Stall hygiene score	0.119	4.54
Udder hygiene score	0.643	24.55
Hand milking	−0.756	28.87
Total	2.619	100

SCC=Somatic cell count

The discriminate function fitted was as follows:

D=−3.964+1.150 M1+0.044 M2−0.150 M3+0.186 M6+0.926 M7−1.536 M8

The function fitted demonstrated 89.2% accuracy with p<0.001. Thus, it could be inferred that the variables considered in the present analysis together were able to classify effectively normal and infected animals. The functions at group centroids were −0.982 and 1.209, respectively, for normal and mastitis-infected animals. Centroids are actually the group means. Cases with scores near to a centroid were predicted as belonging to that group.

## Discussion

The findings of ROC curve analysis are in contrast to the findings by other researchers [7-11] and recommendations of the International Dairy Federation [12] who indicated that the mean values of SCC for the sub-clinically affected udder were 500,000 and above cells/ml of milk. The findings also do not match with the findings of Tarate *et al*. [4], Samantal *et al*. [13], De and Mukherjee [14], Elango *et al*. [15], Gera and Guha [16], Singh and Garg [17,18], and Das *et al*. [19] (Table 3). This might be because most of the earlier findings rely on international standards laid by IDF in 1971. However, a revised study to define the threshold SCC count to differentiate subclinical cases from the normal ones using advanced techniques like ROC curve needs to be carried out.

**Table-3:** Reported values of SCC in milk of animals suffering from subclinical mastitis.

Sr No.	Species	Breed/Species	SCC (lakh cells/ml)	References
1	Buffalo	Murrha	7.2±0.3	[22]
2	Indigenous Cattle	Sahiwal	6.8±0.2	[22]
3		Kankrej	2.42	[4]
4		Gir	2.22	[4]
5	Crossbreed cattle	Karan Fries	8.3±0.69	[13]
6		Karan Swiss	7.2±0.7 11.28±0.92	[13,14]
7		HF×Brown Swiss×Hariyana	10.54±0.7	[14]
8		HF×Hariyana	15.51±0.94	[14]
9		Crossbreed	3.58−4.04	[18]
10		Crossbreed	2.34±0.44	[16]

SCC=Somatic cell count

In a study conducted by Petzer *et al*. [20] in South Africa, for the cutoff level of 150,000 cells/mL, sensitivity in composite milk samples was 65.3% and specificity was 66.8% on conducting ROC curve analysis. The area under the curve of the ROC graph was 0.7084, indicating that the SCC test could be considered as a good indicator of IMI. The sensitivity and specificity values in our study are considerably high (92.6% and 91.5%, respectively) at the threshold of 310,000 cells/ml of milk, though the sample size studied is comparatively small.

Thirunavukkarasu [21] studied a discriminate model and reported that average daily milk yield contributed the maximum for differentiating the normal and mastitis-infected animals. In the present study, however, test day milk yield was not found to contribute significantly. Udder hygiene score was found to contribute maximum in both the studies. Stage of lactation and season of collection were also found to be significantly contributing to the total distance measured for discriminating normal and infected animals. A highly significant classificatory variable, i.e., SCC was introduced in the present model which was not considered by Thirunavukkarasu [21].

## Conclusion

Fanatical monitoring of milk SCC can help the livestock owners to set the alarm for entry of IMI in the herd. However, since the cells are shed in milk regularly in a limited number, knowing the cutoff to differentiate the normal from the subclinical must be known. ROC curve analysis performed in the present study represented that 93% of the case could be correctly predicted as mastitis infected using SCC as a marker (p<0.001). The study also indicated that 310,000 somatic cells/ml of milk should be the threshold value to differentiate the subclinically affected animals from the normal ones. The discriminate function fitted in the present study demonstrated 89.2% accuracy with p<0.001. The model is designed to be more field applicable using the data on SSC count and management conditions. Both the functions can be effectively used for delineation of subclinical mastitis cases.

## Author’s Contributions

PVJ collected the data and the samples from the field. DND planned the experiment, and this work was inspired by BRS. KPS performed statistical analysis. BRS provided the primer information and standardization bacterial isolation from milk. PVJ did sample processing and compiling and perpetration of the draft for the paper. All authors read and approved the final manuscript.
